# IMPACT study: Impact of adherence to anti‐VEGF intravitreal injections for macular disease during COVID 19‐related confinement in France

**DOI:** 10.1111/aos.15206

**Published:** 2022-06-29

**Authors:** Victoire Hurand, Jean‐Baptiste Ducloyer, Florian Baudin, Serge Aho, Michel Weber, Laurent Kodjikian, François Devin, Pierre‐Henry Gabrielle, Catherine Creuzot‐Garcher, Pascale Massin, for the CFSR Research Net

**Affiliations:** ^1^ Department of Ophthalmology University Hospital Dijon France; ^2^ Department of Ophthalmology University Hospital Nantes France; ^3^ Pathophysiology and Epidemiology of Cerebro‐Cardiovascular Diseases (PEC2) University Hospital Dijon France; ^4^ Department of Epidemiology University Hospital Dijon France; ^5^ Department of Ophthalmology Croix Rousse University Hospital Lyon 4 France; ^6^ Center of Ophthalmology Monticelli Paradis Marseille France; ^7^ Eye and Nutrition Research Group GSGA, INRAe Burgundy, Dijon France; ^8^ Center of Ophthalmology Breteuil Paris France

**Keywords:** COVID‐19, visual acuity, nAMD, intravitreal injection

## Abstract

**Purpose:**

The aim of this study was to evaluate the impact of adherence to French coronavirus disease 2019 (COVID 19)‐related guidelines for intravitreal injection (IVI) practice on the visual outcomes of patients treated with anti‐vascular endothelial growth factor (VEGF) agents for macular diseases during the first lockdown period.

**Methods:**

Observational multicentre study including all patients from 18 centres with an IVI initially planned during the lockdown. Visual acuity (VA, ETDRS) was recorded at 1 and 4 months after lockdown. French COVID 19‐related guidelines recommended maintaining IVI practice. We defined three groups of patients: A, adherent to guidelines; NA+, non‐adherent with delayed IVIs; and NA−, non‐adherent without IVIs performed during the lockdown. Risk factors for non‐adherence and visual loss were studied.

**Results:**

A total of 3020 eyes of 3020 patients, aged 77.8 ± 11.6 years, 59.8% women, were included. 59.3% were non‐adherent(46.7% NA+, 12.6% NA−). A smaller decrease in VA at 4 months was observed in the A group than the NA+ and NA− group (−0.2 ± 6.7, −0.3 ± 6.9 and −1.5 ± 6.9, respectively [*p* < 0.001]). Factors associated with non‐adherence were in multivariable analysis, older age, hospital practice, low‐density population areas, high viral incidence areas, longer intervals between injection and treat and extent protocol. Factors associated with visual loss at 4 months in multivariable analysis were, being in the NA− group, older age, T&E and fixed regimens.

**Conclusion:**

Strict adherence to guidelines was associated with better visual outcome, although most of our patients did not attend as planned. Identification of patients at risk could help in the future in case of a new pandemic lockdown.

## 1 INTRODUCTION

Coronavirus 2019 (COVID‐19) infection, responsible for the severe acute respiratory syndrome (SARS‐CoV2), was first identified in December 2019 (Petersen et al., 2020). The World Health Organization (WHO) initially declared it as a Public Health Emergency of International Concern on 30 January 2020, and officially confirmed the COVID‐19 as a pandemic outbreak on 11 March 2020. In France, the entire country was kept under lockdown for 8 weeks, from 17 March to 11 May 2020 (Légifrance, 2020), with restrictions on movement except for essential needs, work and health‐related outings.

The coronavirus, being of air transmission, quickly appeared that certain specialties could be at increased risk of contamination due to close contact and a high volume of patients (Breazzano et al., 2020). Therefore, national societies of ophthalmology issued guidelines to limit the risks of viral infection. The aim of intravitreal injections (IVI) was to accommodate both risk reductions for patients under treatment and to minimize the time spent for IVI treatments (Lim et al., 2020; The Royal College of Ophthalmologists, COVID‐19 review team, 2020). In many countries, prior to performing IVI, visual acuity (VA) assessment, optical coherence tomography (OCT) and fundus ocular examination were no more performed if not strictly necessary (Colantuono et al., 2020).

The French Society of Ophthalmology (Société Française ‘d'Ophtalmologie [SFO]) provided guidelines via its website and emails for ophthalmologists advising, first, to maintain IVI to avoid vision loss, particularly in patients with neovascular age‐related macular degeneration (nAMD), and then to apply the same interval as before lockdown without any visual acuity or retinal imaging examination (Kodjikian, 2020).

However, as many patients did not show up, the French club of retinal physicians (Club Francophone des Spécialistes de la Rétine [CFSR]) decided to conduct a study among their members to evaluate the impact of lockdown on visual outcomes in patients treated with IVI of anti‐vascular endothelial growth factor (VEGF) agents for macular diseases. Our first objective was to measure the effect of SFO guidelines on patient visual outcomes 4 months after lockdown. Secondary objectives were to assess the percentage of patients who followed SFO guidelines in different macular diseases and identify the risk factors of poor adherence to the IVI planned regimen and the risk factors of visual loss.

## 2 MATERIAL AND METHODS

### 2.1 Design of the study

The SFO guidelines were published on 18 March 2020, following the strict French lockdown from 17 March to 11 May 2020. It defined the following statements: for patients with neovascular age‐related macular degeneration, considering that a regular schedule of anti‐VEGF injections is a key issue to stabilize vision, the guidelines were to apply the same interval as observed before, during the lockdown period. For instance, a patient injected with a 6‐week interval before the lockdown was injected at a similar fixed interval during lockdown without any functional or anatomical examination. The patient was then advised to return 6 weeks later for another injection. Regarding diabetic macula oedema (DME) or macular oedema secondary to retinal vein occlusion (RVO), the guidelines were to postpone IVI. However, most centres decided to apply the nAMD guidelines to all macular diseases and maintained a fixed injection regimen during lockdown. Private and public centres and members of the CFSR were contacted to participate in this observational study. All participating centres agreed to fill out a standardized questionnaire to record characteristics of patients whom injection was planned during lockdown (whether performed or not). Then, ophthalmologists collected data about those patients at 4‐month post‐lockdown. The protocol followed the tenets of the declaration of Helsinki. The local institutional review board (Comité de protection des personnes, region Est) approval was not required because of our study's non‐interventional and retrospective design. This study was registered on clinical trial.gov (NCT04395859).

### 2.2 Study population

Data from all patients scheduled for IVI of VEGF inhibitors during the French lockdown from 17 March to 11 May 2020 were collected from 18 centres in France (9 in hospital and 9 in private practice). Patients who were not scheduled to be injected during this period were not included in the present study. Pre‐lockdown data collected were general clinical characteristics, type of macular diseases (namely nAMD, DME, RVO, myopic choroidal neovascularization [mCNV] and polypoidal vasculopathy or other causes), treatment regimen, last recorded IVI interval before lockdown, last recorded VA (ETDRS scale and Early Treatment of Diabetic Retinopathy Study). Then, investigators were asked to collect data on the delay between initially planned and the real timing of the injection during the lockdown and VA at 4 (±1)‐month post‐lockdown. In case of bilateral involvement, one eye per patient was randomly selected and included for analysis. Local COVID‐19 incidence rates attributed to practice were extracted from Santé Publique France data on 15 March 2020 (France, 2020). Areas were defined as those with low viral incidence if less than 5 cases for 100 000 inhabitants, contrary to a high incidence rate with 5 or more cases for 100 000 inhabitants (France, 2020).

### 2.3 Definitions

We defined three groups according to SFO guideline adherence: strict adherence (*Adherence group, A*), that is strictly maintaining the intervals as observed before lockdown (±1 week). All patients who postponed an IVI to the one initially planned were considered as non‐adherent. We divided this group in 2 according to the realization or not of an IVI during the lockdown: non‐strict adherence, that is with a longer delay to IVIs than observed before lockdown (*Non‐Adherence group with IVI with a delay > 1 week, NA+*) and finally non‐adherence with a complete therapeutic break, that is without carrying out any injection during lockdown (*Non‐Adherence group without IVI, NA−*). We aimed to distinguish patients who did not have an injection from those who had a delayed injection. We based our analysis on the schedule of planned injections and not on the schedule of honoured injections to highlight rescheduling.

### 2.4 Main outcome measures

The main objective was to measure the effect of lockdown and SFO guidelines adherence on VA change at 4 (±1) months. The secondary objectives were (1) to assess the influence of macular disease aetiology on the visual outcome, depending on adherence with guidelines and (2) to identify the risk factors for IVI poor adherence and visual loss.

### 2.5 Statistical analysis

Qualitative variables were expressed in terms of counts (percentages). Confidence intervals were estimated using an exact binomial method. Quantitative variables were described by their mean, standard deviation, median and interquartile range. Relationships between a quantitative outcome and its predictors (covariates) were studied using simple linear regression (univariable analysis) or multiple linear regression (multivariable analysis). Relationships between a binary outcome and its covariates were studied using bivariable or multivariable logistic regression, expressed as relative risk (RRR). A robust variance estimator was used (Herbert & Kott, 2006). Non‐linear relationship between outcome and continuous covariates was carried out using fractional polynomials (Royston et al., 1999). Mixed models were used to account for potential centre‐effect (Kahan, 2014). Multiple imputations accounted for missing data by chained equations (with 20 imputed datasets) (White et al., 2011). Graphical and numerical diagnostic tools were used to check multiple imputations. The goodness of fit and discrimination was evaluated using the Hosmer–Lemeshow test via ROC curves for logistic regression. Internal validation of regression models was performed using bootstrap (3000 replicates) (Steyerberg et al., 2001). Statistical analyses were performed using Stata (StataCorp. 2017. Stata Statistical Software: Release 15. College Station, TX: StataCorp LLC.) (Stata, 2017).

## 3 RESULTS

Charts from 3020 patients representing 3020 eyes were collected during the study period with a mean ± SD age of 77.8 ± 11.6 years; 59.8% were women. The characteristics of the population are summarized in Table 1. Mean VA before the lockdown was 64.5 ± 18.4 ETDRS letters. The mean interval between injections before the lockdown was 7.4 ± 4.9 weeks. A total of 2030 eyes (67.7%) were treated for nAMD. The majority of the patients were injected using a treat & extend (T&E) regimen before the lockdown, while 306 (10.4%) patients were in the induction phase of the treatment (Table 1). The rate of strict adherence to French guidelines (A) was 40.7% (1230 patients), vs. non‐adherence in 59.3% (NA+ and NA−) (1790 patients). The characteristics of the population according to the SFO guideline adherence groups are presented in Table 2. From the 3020 eyes who had an IVT planned a priori during the lockdown, 2345 eyes only were examined at least once during the 4‐month follow‐up. There was no difference in pre‐lockdown VA among the three groups. NA− patients without IVI during lockdown were significantly older and more likely to be living in areas with a high incidence of COVID‐19 than A patients (*p* < 0.001 and *p* < 0.001, respectively). Moreover, patients in the NA− group were more often living in densely populated areas than the two other groups, NA+ and A (*p* < 0.001). Patients with a longer mean injection interval before lockdown were more prone to postpone the following IVI and be non‐adherent (A group, mean ± SD injection interval before the lockdown of 6.9 ± 3.0 weeks; NA+ group, 7.5 ± 4.9 weeks; NA− group, 8.3 ± 8.5 weeks [*p* = 0.005]).

**Table 1:** Population characteristics (*N* = 3020 eyes) for 3020 patients

	General population
Group (*N* = 3020) (%)	
A group	1230 (40.7%)
NA+ group	1411 (46.7%)
NA− group	379 (12.6%)
Sex (*N* = 2930) (%)	
Women	1751 (59.8%)
Men	1179 (40.2%)
Age, mean ± SD (years)	77.8 ± 11.6
Age (*N* = 2919) (%)	
≤72	809 (27.7%)
73–80	723 (24.8%)
81–87	811 (27.8%)
≥ 88	576 (19.7%)
Eye (*N* = 3017) (%)	
Right	1543 (51.1%)
Left	1474 (48.9%)
Pathology (*N* = 2997) (%)	
nAMD	2030 (67.7%)
DME	423 (14.1%)
RVO	350 (11.8%)
Other	112 (3.7%)
mCNV	52 (1.7%)
Polypoidal vasculopathy	30 (1.0%)
Viral incidence by area (*N* = 3020) (%)	
High incidence (Dijon, Lyon, Grenoble, Paris, Marseille, Montpellier)	2143 (71.0%)
Low incidence (Perpignan, Quimper, Brest, Tours, Rouen, Nantes)	877 (29.0%)
Type of practice (*N* = 2943) (%)	
Hospital	1821 (61.9%)
Private practice	1122 (38.1%)
Population density (inhabitants/km^2^) (*N* = 2943) (%)	
<7000	1982 (67.4%)
>7000	961 (32.6%)
Protocol before the lockdown (*N* = 2954) (%)	
Induction phase	306 (10.4%)
T&E	1502 (50.8%)
Fixed regimen	830 (28.1%)
PRN	316 (10.7%)
IVI interval before lockdown, mean ± SD (weeks)	7.4 ± 4.9
Visual acuity before lockdown, mean ± SD (ETDRS)	64.5 ± 18.4

*Note*: Mean ± standard deviation for continuous variables.

Numbers (percentage) for qualitative variables.

Abbreviations: A, Adherence group; DME, Diabetic macular oedema; mCNV, Myopic choroidal neovascularization; NA−, Non‐adherence group without IVI; NA+, Non‐adherence group with IVI; nAMD, Neovascular age‐related macular degeneration; PRN, ProReNata; RVO, Macular oedema secondary to retinal vein occlusion; T&E, Treat & extend.

**Table 2:** Characteristics depending on adherence to French guidelines

	A group *N* = 1230 patients (40.7%)	NA+ group *N* = 1411 patients (46.7%)	NA− group *N* = 379 patients (12.6%)	*p*‐Value
Sex (*N* = 2930)				0.33
Woman	703 (58.9%)	813 (59.6%)	235 (63.2%)	
Age (years) (*N* = 2919)				**<0.001**
≤72	354 (29.4%)	383 (28.3%)	72 (19.8%)	
73–80	317 (26.4%)	346 (25.5%)	60 (16.6%)	
81–87	325 (27.1%)	371 (27.4%)	115(31.8%)	
≥88	206 (17.1%)	255 (18.8%)	115 (31.8%)	
Eye (*N* = 3017)				0.72
Right	637 (51.8%)	710 (50.3%)	196 (51.7%)	
Pathology (*N* = 2996)				0.37
nAMD	830 (68.0%)	935 (66.9%)	264 (70.1%)	
DME	165 (13.5%)	207 (14.8%)	51 (13.5%)	
RVO	140 (11.5%)	162 (11.6%)	48 (12.7%)	
Other	46 (3.8%)	54 (3.8%)	12(3.2%)	
mCNV	26 (2.1%)	24 (1.7%)	2 (0.5%)	
Polypoidal vasculopathy	13 (1.1%)	17 (1.2%)	0	
Viral incidence by area (*N* = 3020)				**<0.001**
High incidence (Dijon, Lyon, Grenoble, Paris, Marseille, Montpellier)	877 (71.3%)	961 (68.1%)	305 (80.5%)	
Low incidence (Perpignan, Quimper, Brest, Tours, Rouen, Nantes)	353 (28.7%)	450 (31.9%)	74 (19.5%)	
Type of practice (*N* = 2943)				**<0.001**
Hospital	829 (68.5%)	734 (53.8%)	258 (69.7%)	
Private practice	381 (31.5%)	629 (46.2%)	112 (30.3%)	
Population density by city (inhabitants/km^2^) (*N* = 2943)				**<0.001**
<7000	883 (73.0%)	903 (66.3%)	196 (53.0%)	
>7000	327 (27.0%)	460 (33.7%)	174 (47.0%)	
Protocol (*N* = 2954)				**<0.001**
Induction phase	134 (11.0%)	140 (10.0%)	32 (9.3%)	
T&E	691 (57.0%)	648 (46.3%)	163 (47.7%)	
Fixed regimen	314 (26.0%)	431 (30.8%)	85 (24.8%)	
PRN	73 (6.0%)	181 (12.9%)	62 (18.2%)	
IVI interval before lockdown (weeks) (*N* = 2971)	6.9 ± 3.0	7.5 ± 4.9	8.3 ± 8.5	**0.005**
Visual acuity before lockdown (ETDRS) (*N* = 2914)	64.3 ± 18.6	65.0 ± 18.1	63.1 ± 18.9	0.14

*Note*: Continuous variables are displayed as mean ± standard deviation. Categorical variables are displayed as number (percentage). Statistically significant *p*‐values are in bold.

Abbreviations: A, Adherence group; DME, Diabetic macular oedema; mCNV, Myopic choroidal neovascularization; NA+, Non‐adherence group with IVI; NA−, Non‐adherence group without IVI; nAMD, Neovascular age‐related macular degeneration; PRN, ProReNata; RVO, Macular oedema secondary to retinal vein occlusion; T&E, Treat & extend.

Visual acuity was measured at 4 months in 2345 patients with 22% of patients who did not show up within the 4‐month post‐lockdown. Overall, for those who attended a visit, the mean VA change at 4 months after the lockdown was −0.4 ± 6.8 ETDRS letters with a mean VA at 4 months of 64.7 ± 18.9 ETDRS letters. We found a significant difference in absolute VA between groups at 1 and 4 months after lockdown, with 64.6 ± 18.9 ETDRS letters for A group vs. 60.4 ± 19.5 for NA− group at 1 month, and 64.9 ± 19.0 vs. 61.4 ± 20.5 at 4 months (*p* < 0.001, *p* = 0.03, respectively). The NA− group (−1.5 ± 6.9 letters) had significantly lower mean VA change 4 months after lockdown than the NA+ group (−0.3 ± 6.9 letters) and the A group (−0.2 ± 6.7 letters) (*p* < 0.001). The visual outcomes at 1 and 4 months after lockdown according to the type of macular diseases are shown in Table 3. Concerning macular diseases, we observed a sharper decrease in VA at 4 months in patients with nAMD and polypoidal vasculopathy, −0.8 ± 6.9 and −2.5 ± 4.2 letters, respectively. On the contrary, patients with RVO increased their VA at 4 months, 0.9 ± 6.4 letters (*p* = 0.008), while no significant difference was observed for DME, 0.4 ± 6.6 letters (*p* = 0.28).

**Table 3:** Visual acuity at 1 and 4 months after lockdown, ETDRS

	A group	NA+ group	NA− group	Total	*p*‐Value
Visual acuity before lockdown (*N* = 2914)	64.3 ± 18.6	65.0 ± 18.1	63.1 ± 18.9	64.5 ± 18.4	0.14
Visual acuity before lockdown according to pathologies (*N* = 2895)					**0.01**
nAMD				63.8 ± 18.8	
DME				66.8 ± 17.1	
RVO				65.5 ± 17.7	
Other				65.1 ± 17.8	
mCNV				60.5 ± 18.8	
Polypoidal vasculopathy				69.5 ± 15.2	
Visual acuity at 1 month after lockdown (*N* = 2540)	64.6 ± 18.9	64.7 ± 18.8	60.4 ± 19.5	64.2 ± 19.0	**<0.001**
Visual acuity at 1 month according to pathologies (*N* = 2531)					**0.005**
nAMD	64.1 ± 18.7	63.9 ± 18.9	59.1 ± 20.3		
DME	67.5 ± 19.4	66.7 ± 17.0	71.6 ± 11.5		
RVO	65.7 ± 21.2	66.5 ± 18.6	55.0 ± 17.4		
Other	64.7 ± 15.6	65.2 ± 23.0	69.6 ± 14.8		
mCNV	63.8 ± 16.0	58.8 ± 23.6	80.5 ± 6.4		
Polypoidal vasculopathy	57.9 ± 17.3	73.5 ± 12.0	‐		
Visual acuity at 4 months after lockdown (*N* = 2345)	64.9 ± 19.0	65.2 ± 18.4	61.4 ± 20.5	64.7 ± 18.9	**0.03**
Visual acuity change at 4 months after lockdown	−0.2 ± 6.7	−0.3 ± 6.9	−1.5 ± 6.9	−0.4 ± 6.8	**<0.001**
Visual acuity at 4 months according to pathologies (*N* = 2329)					**0.04**
nAMD	64.3 ± 18.7	64.2 ± 18.8	59.7 ± 21.2		
DME	67.3 ± 18.8	67.5 ± 15.8	71.9 ± 12.6		
RVO	66.4 ± 21.7	67.6 ± 17.4	59.3 ± 21.0		
Other	64.4 ± 18.1	66.5 ± 21.0	67.7 ± 16.3		
mCNV	64.0 ± 17.5	59.4 ± 21.6	‐		
Polypoidal vasculopathy	57.4 ± 17.8	72.3 ± 16.4	‐		
Visual acuity change at 4 months according to pathologies					**<0.001**
nAMD				−0.8 ± 6.9	Ref
DME				0.4 ± 6.6	0.28
RVO				0.9 ± 6.4	**0.008**
Other				0.9 ± 6.8	0.15
mCNV				0.4 ± 7.2	0.70
Polypoidal vasculopathy				−2.5 ± 4.2	0.08

*Note*: Continuous variables are displayed as mean ± standard deviation. Statistically significant *p*‐values are in bold.

Abbreviations: A, adherence group; DME, diabetic macular oedema; mCNV, myopic choroidal neovascularization; NA+, non‐adherence group with IVI; NA−, non‐adherence group without IVI; nAMD, neovascular age‐related macular degeneration; RVO, macular oedema secondary to retinal vein occlusion.

Table 4 reports the multivariable analysis of factors associated with SFO guidelines adherence. Factors associated with non‐adherence (combining NA+ and NA− groups) vs. A group were older age (RRR 1.56, 95% CI [1.17–2.09], *p* = 0.002), being treated in hospital (RRR 1.92, 95% CI [1.58–2.34], *p* < 0.01), T&E protocol (RRR 1.95, 95% CI [1.45–2.61], *p* < 0.001), living in a area of high viral incidence (RRR 1.60, 95% CI [1.30–1.98], *p* < 0.001) and longer interval (RRR 0.96, 95% [0.94–0.98], *p* < 0.001). Patients living in densely populated areas were less likely to be adherent than those living in low‐populated areas (RRR 0.42, 95% CI [0.34–0.52], *p* < 0.001). The type of macular disease was not associated with the risk of being non‐adherent.

**Table 4:** Multivariable analysis of factors associated with non‐adherence to French guidelines (*N* = 2664 eyes)

	A versus (NA+ + NA−) (ref)	
	RRR (95% CI)	*p*‐Value
Sex (reference = women)		
Men	1.00 (0.85–1.18)	0.99
Age (reference = ≤72 years)		
73–80	0.94 (0.74–1.21)	0.65
81–87	1.25 (0.98–1.61)	0.07
≥88	1.56 (1.17–2.09)	**0.002**
Eye (reference = right)		
Left	0.88 (0.75–1.04)	0.13
Type of practice (reference = private practice)		
Hospital	1.92 (1.58–2.34)	**<0.001**
Viral incidence by area (reference = low incidence)		
High incidence	1.60 (1.30–1.98)	**<0.001**
Pathology (reference = nAMD)		
DME	0.87 (0.66–1.17)	0.35
RVO	0.91 (0.68–1.20)	0.50
Other	0.85 (0.55–1.33)	0.48
mCNV	1.17 (0.63–2.18)	0.61
Polypoidal vasculopathy	0.82 (0.40–1.68)	0.59
Protocol (reference = induction)		
T&E	1.95 (1.45–2.61)	**<0.001**
Fixed regimen	1.35 (0.98–1.85)	0.07
PRN	0.59 (0.38–0.91)	**0.02**
IVI interval before lockdown	0.96 (0.94–0.98)	**<0.001**
Visual acuity before lockdown	1.00 (0.99–1.01)	0.66
Population density (reference = <7000 inhabitants/km^2^)		
>7000 inhabitants/km^2^	0.42 (0.34–0.52)	**<0.001**

*Note*: Statistically significant *p*‐values are in bold.

Abbreviations: A, adherence group; CI, confidence interval; DME, diabetic macular oedema; mCNV, myopic choroidal neovascularization; NA+, non‐adherence group with IVI; NA−, non‐adherence group without IVI; nAMD, neovascular age‐related macular degeneration; PRN, ProReNata; RRR, relative risk ratio; RVO, macular oedema secondary to retinal vein occlusion; T&E, treat & extend.

Table 5 reports the multivariable analysis of factors associated with VA loss at 4 months. Being in NA− group (*p* = 0.01), older age (*p* < 0.01) were found as significantly associated with VA loss at 4 months. Patients treated with T&E protocol and fixed regimen were at higher risk of VA loss (*p* = 0.05, *p* = 0.04, respectively) than patients treated with monthly regimen. RVO patients were significantly less likely to have a visual loss at 4 months than nAMD patients (*p* = 0.01). Finally, patients with good VA before lockdown period had more risk to have a visual loss 4 months after lockdown (*p* < 0.01).

**Table 5:** Multivariable analysis of factors associated with visual loss at 4 months (*N* = 2140 eyes)

	Beta coefficient (95% CI)	p‐value
Adherence (reference = A group)		
NA+ group	−0.16 (−0.77–0.44)	0.60
NA− group	−1.42 (−2.46 – −0.37)	**0.01**
(NA+ + NA−) groups	0.36 (−0.23–0.95)	0.23
Sex (reference = women)		
Men	−0.07 (−0.66–0.52)	0.82
Age (reference = ≤ 72 years)		
73–80	−0.35 (−1.14–0.44)	0.40
81–87	−1.59 (−2.42 – −0.77)	**<0.001**
≥88	−0.98 (−1.95–0.17)	**0.05**
Type of practice (reference = private practice)		
Hospital	−0.03 (−0.67–0.60)	0.92
Viral incidence by area (reference = low incidence)		
High incidence	0.10 (−0.63–0.83)	0.79
Pathology (reference = nAMD)		
DME	0.53 (−0.43–1.49)	0.28
RVO	1.16 (0.24 – −2.09)	**0.01**
Other	1.04 (−0.41 – −2.49)	0.16
mCNV	0.28 (−2.06 – −2.62)	0.81
Polypoidal vasculopathy	−1.60 (−3.35–0.15)	0.07
Protocol (reference = induction)		
T&E	−1.14 (−2.26 – −0.02)	**0.05**
Fixed regimen	−1.21 (−2.39 – −0.03)	**0.04**
PRN	−0.80 (−2.35–0.75)	0.31
IVI interval before lockdown	−0.01 (−0.08–0.59)	0.76
Visual acuity before lockdown	−0.05 (−0.06 – −0.03)	**<0.01**
Population density (reference = <7000 inhabitants/km^2^)		
>7000 inhabitants/km^2^	0.17 (−0.58–0.92)	0.66

*Note*: Statistically significant *p*±values are in bold.

Abbreviations: A, adherence group; CI, confidence interval; DME, diabetic macular oedema; mCNV, myopic choroidal neovascularization; NA+, non‐adherence group with IVI; NA−, non‐adherence group without IVI; nAMD, neovascular age‐related macular degeneration; PRN, ProReNata; RRR, relative risk ratio; RVO, macular oedema linked to retinal vein occlusion; T&E, treat & extend.

## 4 DISCUSSION

Our study reported a relatively low rate of adherent patients (40.7%) to the SFO IVI guidelines during the lockdown. Most of our patients either delayed or did not receive their IVI during the lockdown. Poor adherence was associated with a higher risk of visual loss, especially for those that did not receive IVI during lockdown with a mean visual loss of −1.5 ± 6.9 ETDRS letters at 4‐month post‐lockdown.

In multivariable analysis, significant factors associated with visual loss 4 months after lockdown were non‐adherence, older age, both T&E and fixed regimens, and better VA before lockdown. By contrast, RVO patients were significantly less likely to have a visual loss at 4 months after lockdown than nAMD patients. Non‐adherence and older age are well known factors for visual loss in nAMD patients, although a delay in injection schedule is associated with greater visual loss (Muether et al., 2011). The good visual outcomes for RVO patients need to be confirmed since this type of retinal disease represented only 10% of the population.

A drop‐in attendance was observed globally during the lockdown, with a significant reduction in the number of ophthalmic procedures (Corradetti et al., 2020; Toro et al., 2021). Ophthalmology was one of the specialties with the most significant patient volume reduction due to the COVID‐19 pandemic (strata, 2020). Concerning IVI, a significant decrease was found in April 2020 with a drop in the number of IVI of 38.6% in 17 US institutions (Breazzano et al., 2021). Our results are similar to those observed by Song et al., (2021). on patient adherence to protocol during the pandemic in the United States (Ohio). They identified that 40.7% of the patients received their IVI at the planned interval, 46.7% postponed their injection, and 12.6% did not receive any injection during the lockdown. The authors reported that the decrease in IVI was prolonged after lockdown (Billioti de Gage et al., 2021), but it could simply be due to a long interval planned before lockdown with no scheduled injection during or after this period.

Several studies on COVID‐19 impact found that patients with missing visits would lose more vision than those who attended. Song et al. defined three categories of patients: ‘completers’ if the patient attended visit, ‘cancelers’ if cancellation was documented before the visit or ‘no‐show’ if the patient did not check‐in for the scheduled visit and did not provide prior notification. Comparably to our finding, they showed that no‐show patients lost more vision than others, −5.0 ± 1.9 letters; vs. −1.6 ± 0.7; and 0.4 ± 0.5, for cancellers and completers, respectively, the latter maintaining VA (Song et al., 2021).

Similarly, Narvane et al. observed in the United States (Minnesota) that patients who delayed their injection appointment by more than 2 weeks had a visual loss after the post‐lockdown period (Naravane et al., 2021). However, these results raise concerns about irreversible vision loss. Indeed, our study collected data about patients who finally had a visit after lockdown with VA measurement, although delayed. Unfortunately, we could not measure the post‐lockdown VA from the lost to follow‐up patients who did not attend any appointment after lockdown. From the 3020 eyes who had an IVT planned during the lockdown, 2345 (78% of patients) were considered for VA analysis at 4‐month post‐lockdown with 22% no‐show patients during the 4‐month follow‐up. The characteristics of the patients lost to follow‐up can be found in Appendix S1. It is very likely that patients lost to follow‐up who did not receive any treatment experienced an even higher visual loss.

In our study, poor adherence led to visual loss, especially in patients with polypoidal vasculopathy. Our results differed from those of Song et al., (2021), who found that both RVO and DME were associated with the most significant decrease in VA after lockdown, −3.5 ± 1.9 and −3.2 ± 1.4 letters, respectively. The main difference between these two studies was the delay for VA assessment after lockdown, which was not set in Song et al.’s study.

According to retinal diseases, the difference in guidelines between countries may explain the discrepancy in visual outcomes between studies. The Royal College of Ophthalmologists, COVID‐19 review team (2020), in its ‘Retinal Medical Management Plans during COVID‐19’ protocol, made a distinction between ‘already under review’ and ‘new’ patients. For ‘already under review’ nAMD patients, they recommended maintaining all patients on 8 weeks of anti‐VEGF IVI without clinical review. On the contrary, they postponed anti‐VEGF therapy for ‘already under review’ and ‘new’ DME, RVO for 4 months. The Vision ‘Academy's Steering Committee of international retinal disease experts also provided complete guidance for ophthalmologists on how to deliver the best possible care for patients while minimizing the risk of infection (Korobelnik et al., 2020). As in France, the recommendation was to continue IVIs for patients with nAMD and newly diagnosed retinal pathology unless the patient had risk factors for severe COVID‐19.

In our study, risk factors for non‐adherence were older age, hospital practice, high viral incidence areas, high‐density areas, longer mean injection interval and T&E protocol. Older patients were more likely to miss their injection appointments. This finding is not restricted to pandemic conditions: older age was previously identified as a factor associated with non‐adherence. Ehlken et al., (2018) reported a significant proportion of elderly patients not adhering or not attending to consultations in everyday, non‐epidemic situations. Boulanger‐Scemama et al., (2015) also demonstrated that older patients were significantly associated with loss to follow‐up.

Moreover, Westborg & Rosso, (2018) found an increased non‐adherence to treatment protocol in patients with comorbidity compared with patients without (OR 1.27, 95% CI [1.13–1.43]). It is likely that older patients, who are at higher risk for severe COVID‐19 and potentially have other comorbidities, decided to limit their interactions for fear of contracting the virus (Chen et al., 2020). Furthermore, patients treated in hospitals were more likely to be non‐adherent than those followed in private practice, which could be related to the higher incidence rate of COVID‐19 in hospitals and the fear of contracting it. These results are consistent with Harrison et al.’s, (2021) study that demonstrated increased transport refusals to hospitals during COVID‐19. Moreover, patients living in densely populated areas were less adherent to guidelines, probably trying to limit their movements because of the higher risk of transmission, although a shorter distance to reach the centre can favour adherence (Droege et al., 2013). Recently, new studies did not show any demographic difference between delayed and non‐delayed patients during the COVID‐19 pandemic (Breazzano et al., 2021; Stone et al., 2021). Concerning IVIs protocol, we found that T&E protocol increased the risk for non‐adherence. Usually, fixed or T&E regimens tend to decrease undertreatment compared with PRN regimen. It is possible that patients were more reluctant to come for injection than just a visit. Moreover, the duration of the disease before inclusion was not recorded. Thus, it is possible that some cases from PRN regimen groups were recent diseases with highly motivated patients in the observed and planned period. By contrast, more chronic diseases are usually treated with a fixed or T&E regimen. Other factors can influence protocol adherence, like comorbidities regardless of COVID‐19. Indeed in interviews with non‐adherent patients, comorbidity is often cited as contributing to non‐adherence (Polat et al., 2017). However, these data were not recorded nor ‘patients' status concerning COVID‐19, which could have influenced adherence to guidelines.

We acknowledge some limitations in this study. First, the number of eyes included remains small, representing only 3020 eyes among the 85 000 IVI per month in France, but is one of the largest studies on the impact of lockdown on IVI outcomes recording clinical data with the same recommendations applied in the whole country (Billioti de Gage et al., 2021). Second, this analysis was restricted to the first months following the first French COVID‐19 lockdown and do not necessarily reflect VA's long‐term change. However, the repetition of lockdown after the study period limits the validity of such an analysis. Third, the results of our study cannot be extrapolated to patients lost to follow‐up 4 months after the lockdown, who could have significantly reduced their VA. Nevertheless, our results are giving optimistic outcomes on VA after 4 months. Another weakness of our study is that we did not know precisely the percentage of ophthalmologists who scrupulously respected the SFO guidelines, and on which pathologies. The main strength of this study is its multicentric design, including hospital and private practice and the analysis of IVIs regimen. The distinction between NA+ and NA− patients and stratification by maculopathies provides more detailed information on patients at higher risk of non‐adherence and visual impairment.

In conclusion, our French multicentric study found a high proportion of non‐adherent patients to the SFO IVI guidelines during the lockdown. Non‐adherent patients were at greater risk of visual loss, confirming the need to maintain IVIs in the event of a pandemic. This study gives insight into patient profiles at higher risk of non‐adherence and could help identify the target population for preventive information.

## PARTICIPANTS

Pr Catherine Creuzot‐Garcher, Department of Ophthalmology, University Hospital, Dijon, France.

Pr Laurent Kodjikian, Department of Ophthalmology, Croix Rousse University Hospital, Lyon 4, France.

Pr Corinne Dot, Department of Ophthalmology, Desgenettes Military Hospital, Lyon 3, France.

Pr Pascale Massin, Center of Ophthalmology Breteuil, Paris, France.

Dr Aude Couturier, Department of Ophthalmology, Universital Hospital Lariboisière, Paris, France.

Dr Nabil Herda, Department of Ophthalmology, Universital Hospital, Créteil, France.

Dr Marie‐Laure Le Lez, Department of Ophthalmology, University Hospital Bretonneau, Tours, France.

Dr Guylène Le Meur, Department of Ophthalmology, University Hospital, Nantes, France.

Dr Mathilde Gallice, Department of Ophthalmology, University Hospital, Grenoble, France.

Dr François Devin, Center Monticelli Paradis, Marseille, France.

Dr Elodie Labeille, Ophthalmologic Center de la colline, Caluire‐et‐Cuire, France.

Dr Julien Tilleul, Ophthalmologic Center Pôle Vision Pantin, Pantin, France.

Dr Sandrine Allieu, Center Beau soleil, Montpellier, France.

Dr Guilhem Cartry, Perpignan, France.

Dr Valérie Mané‐Tauty, Quimper, France.

Dr Anne Robinet, Brest, France.

Dr Amélie Lecleire Collet, Clinic Mathilde, Rouen, France.

Dr Hassiba Oubraham‐Mebroukine, Montargis, France.

## Supporting information


Appendix S1
