# Supplementary material for: IMPACT study: Impact of adherence to anti‐VEGF intravitreal injections for macular disease during COVID 19‐related confinement in France
Source: Acta Ophthalmol. 2022 Jun 29:10.1111/aos.15206. Online ahead of print. doi: 10.1111/aos.15206 (PMC9350166; doi:10.1111/aos.15206)
Supplement: Supplementary file 1 — Appendix S1 [file AOS-9999-0-s001.docx]

**Appendix 1.** Missing data characteristics (N= 675)

|  |  | p-value |
| --- | --- | --- |
| Sex (N = 675) (%) |  | 0.18 |
| Women | 388 (57.48) |  |
| Men | 287 (42.52) |  |
| Age, mean ± SD (N = 652) (years) | 78.3 ± 11.8 | 0.15 |
| Pathology (N = 668) (%) |  | **0.002** |
| nAMD | 436 (65.27) |  |
| DME | 126 (18.86) |  |
| RVO | 74 (11.08) |  |
| Other | 16 (2.40) |  |
| mCNV | 11 (1.65) |  |
| Polypoidal vasculopathy | 5 (0.75) |  |
| Protocol before the lockdown (N = 627) (%) |  | **< 0.001** |
| Induction phase | 59 (9.41) |  |
| T&E | 220 (35.09) |  |
| Fixed regimen | 221 (35.25) |  |
| PRN | 127 (20.26) |  |
| IVI interval before lockdown, mean ± SD (N = 651) (weeks) | 7.7 ± 6.2 | 0.25 |
| Visual acuity before lockdown, mean ± SD (N = 592) (ETDRS) | 61.1 ± 19.4 | **< 0.001** |
